# Efficacy and safety profile of phosphodiesterase 4 inhibitor in the treatment of psoriasis: A systematic review and meta-analysis of randomized controlled trials

**DOI:** 10.3389/fimmu.2022.1021537

**Published:** 2022-10-10

**Authors:** Qin Kang, Jing-si Chen, Huan Yang

**Affiliations:** ^1^ Department of Health Statistics and Information Management, School of Public Health, Chongqing Medical University, Chongqing, China; ^2^ Department of Dermatology, Children’s Hospital of Chongqing Medical University, National Clinical Research Center for Child Health and Disorders, Ministry of Education Key Laboratory of Child Development and Disorders, Chongqing, China; ^3^ China International Science and Technology Cooperation Base of Child development and Critical Disorders, Chongqing, China; ^4^ Chongqing Key Laboratory of Child Health and Nutrition, Chongqing, China

**Keywords:** psoriasis, PDE4 inhibitor, apremilast, meta-analysis, treatment strategies

## Abstract

**Background:**

Systemic therapy is an important treatment for psoriasis. Phosphodiesterase 4 (PDE4) inhibitors are new candidates for psoriasis therapy.

**Objectives:**

To evaluate the efficacy and safety of PDE4 inhibitors in psoriasis.

**Method:**

Randomized clinical trials with PDE4 inhibitors vs placebos in patients with psoriasis were identified from MEDLINE, Embase, Cochrane Controlled Register of Trials, ClinicalTrials.gov, from inception to July 14, 2022. The study was registered in PROSPERO (CRD42022345700).

**Results:**

18 studies were identified, 9 of which included moderate-to-severe plaque psoriasis, 2 mild-to-moderate plaque psoriasis, and 7 psoriatic arthritis. A total of 6036 patients were included. Only one oral PDE4 inhibitor, apremilast, met the inclusion criteria. Overall, compared with the placebo, apremilast was associated with higher response rates in PASI-75 (RR, 3.22; 95% CI, 2.59-4.01), ScPGA of 0 or 1 (RR, 2.21; 95% CI, 1.69-2.91), PPPGA of 0 or 1 (RR 2.33; 95%CI, 1.16-4.66), and a significant decrease in NPASI (SMD, -0.46; 95% CI, -0.58 to -0.33). There were no significant differences in serious adverse events. Subgroup analyses showed that significantly more patients achieved PASI-75 after 16 weeks of therapy with apremilast of 20 mg bid (RR, 2.82; 95% CI, 2.01-3.95) and 30 mg bid (RR, 4.08; 95% CI, 3.12-5.33). Heterogeneity was not significant across studies.

**Conclusion:**

Apremilast is a safe and effective treatment for plaque psoriasis and psoriatic arthritis, especially for difficult-to-treat sites.

**Systematic review registration:**

https://www.crd.york.ac.uk/prospero, identifier (CRD42022345700).

## Introduction

Psoriasis is a common chronic relapsing non-infectious inflammatory disease. More than 60 million adults and children are affected worldwide (1). The clinical manifestations of psoriasis are extensive and recurring erythematous scales, especially on areas that affect aesthetics and function, such as the head, nails, palms, and soles, which seriously affect the patients’ mental health and quality of life (2). The lack of long-term and effective control means has brought a serious economic burden to individuals and society, and has become a public health problem of great concern (3).

Treatment strategies for psoriasis should take into account its severity, comorbidities, patient economic status, patient preference (oral or subcutaneous injection), and treatment expectations (1, 2, 4). With the exception of moderate to severe psoriasis, patients with mild psoriasis whose symptoms do not improve after topical therapy or who have psoriatic arthritis (PsA), psoriatic nail involvement, or involvement of the hands, feet, genital areas, or scalp may benefit from systemic therapy (5, 6).

Oral traditional systemic drugs used to treat psoriasis include methotrexate, cyclosporine and acitretin, which can exhibit drug-drug interactions and cumulative organ toxicity (7). Although biologics have shown good results in psoriasis, price may limit their use (8). Apremilast is a small molecule oral phosphodiesterase 4 (PDE4) inhibitor, which exerts anti-inflammatory effect by up-regulating the levels of anti-inflammatory cytokines (e.g., IL-10) (9), and reducing the levels of proinflammatory factors (e.g., TNFα and IL-8) (10). It has been approved by the U.S. Food and Drug Administration (FDA) for the treatment of PsA and moderate to severe plaque psoriasis in 2014 (11).

A recently published meta-analysis evaluated the efficacy and safety of apremilast in the treatment of plaque psoriasis. This study included plaque psoriasis only, not other types of psoriasis, such as PsA. Patients with PsA also have psoriatic skin lesions. And this study only assessed general physical signs (Psoriasis Area Severity Index, PASI; Static Physician Global Assessment, sPGA), not localized lesions, such as nail and scalp (12).

Therefore, in order to further understand the application of PDE4 inhibitor in patients with various types of psoriasis, we used meta-analysis to quantitatively synthesize the current evidence on the efficacy and safety of PDE4 inhibitors in the treatment of various psoriasis, thereby providing a higher level of evidence-based medical evidence for clinical decision-making.

## Methods

We registered the protocol in International Prospective Register of Systematic Reviews (PROSPERO CRD42022345700) and followed the standard methodological guidelines for meta-analysis of randomized clinical trials (RCTs) (13) and the Preferred Reporting Items for Systematic reviews and Meta-Analyses guidelines (PRISMA) (14).

### Data sources and search strategy

We conducted searches in three electronic medical databases, including Medline, Embase and Cochrane Controlled Register of Trials (CENTRIL) from inception to July 14th, 2022. We also searched ClinicalTrials.gov for unpublished trials. No restrictions on languages were placed. The search strategies are provided in **eTable 1**.

### Inclusion and exclusion criteria

Two investigators (HY and QK) independently assessed trial eligibility. We included RCTs comparing PDE4 inhibitors with placebo in the treatment of psoriasis, regardless of study design, age, race/ethnicity, dose, treatment duration, disease severity, and type of psoriasis. We excluded non-human studies, conference abstracts, protocols, studies that did not report outcomes of interest.

### Data extraction

Three reviewers (HY, QK, and J-sC) independently extracted the following information: clinical trial registration number, age and number of subjects, type and severity of psoriasis, intervention details, timing of outcomes, and analyzed outcomes. Outcomes included treatment efficacy outcomes, including the percentage of participants who achieved at least 75% improvement in the PASI (PASI-75) from baseline, the percentage of participants who achieved sPGA, Scalp Physician’s Global Assessment (ScPGA), and Palmoplantar Psoriasis Physician Global Assessment (PPPGA) score of clear (0) or almost Clear (1), change from baseline in Nail Psoriasis Severity Index (NPASI), patient’s assessment of pain and pruritus in visual analogue scale (VAS), and Dermatology Life Quality Index (DLQI). And safety measures included the number of participants with any treatment-emergent adverse event (TEAE), any drug-related TEAE, any serious TEAE, and any TEAE leading to drug withdrawal. We had cross-checked the information to ensure accuracy and completeness of the content. Discrepancies in eligibility decisions were discussed at each stage of the process until the reviewers reached consensus.

### Quality of assessment

Two reviewers (HY and QK) independently assessed study quality according to the Cochrane Handbook for Systematic Reviews of Interventions. Disagreements were resolved through discussions and consensus with the third author (J-sC), and a consensus was finally achieved. In addition, we used Begg’s test and Egger test to assess publication bias.

### Statistical analysis

Dichotomous data were calculated by using relative risk (RR) with 95% confidence intervals (CIs). Continuous data were summarized by using the standardized mean difference (SMD) with 95% CIs. Statistical significance was assumed for P < 0.05. Data were pooled by means of meta-analyses carried out on the full intention-to-treat (ITT) population, using the software Review Manager 5.3 and STATA 12.0. When the information was not available, we followed cochrane handbook to calculate the unreported values from the reported pre- and post-intervention means, SDs, number of participants, and standard error.

Heterogeneity was assessed by using the Cochran Q test and quantified with the I^2^ statistic. A p value of 0.10 or less or an I^2^ of 50% or more indicated substantial heterogeneity. If statistical heterogeneity exists, we used sensitivity analyses to investigate the possible causes of heterogeneity and assess the robustness of the results. Subgroup analyses were used to further stratify the trials by psoriasis type, treatment dose, and duration. Based on the assumption that clinical and methodological heterogeneity may exist and influence the results, a random-effects meta-analysis model was performed to pool the data.

## Results

### Results of the search

**Figure 1:**
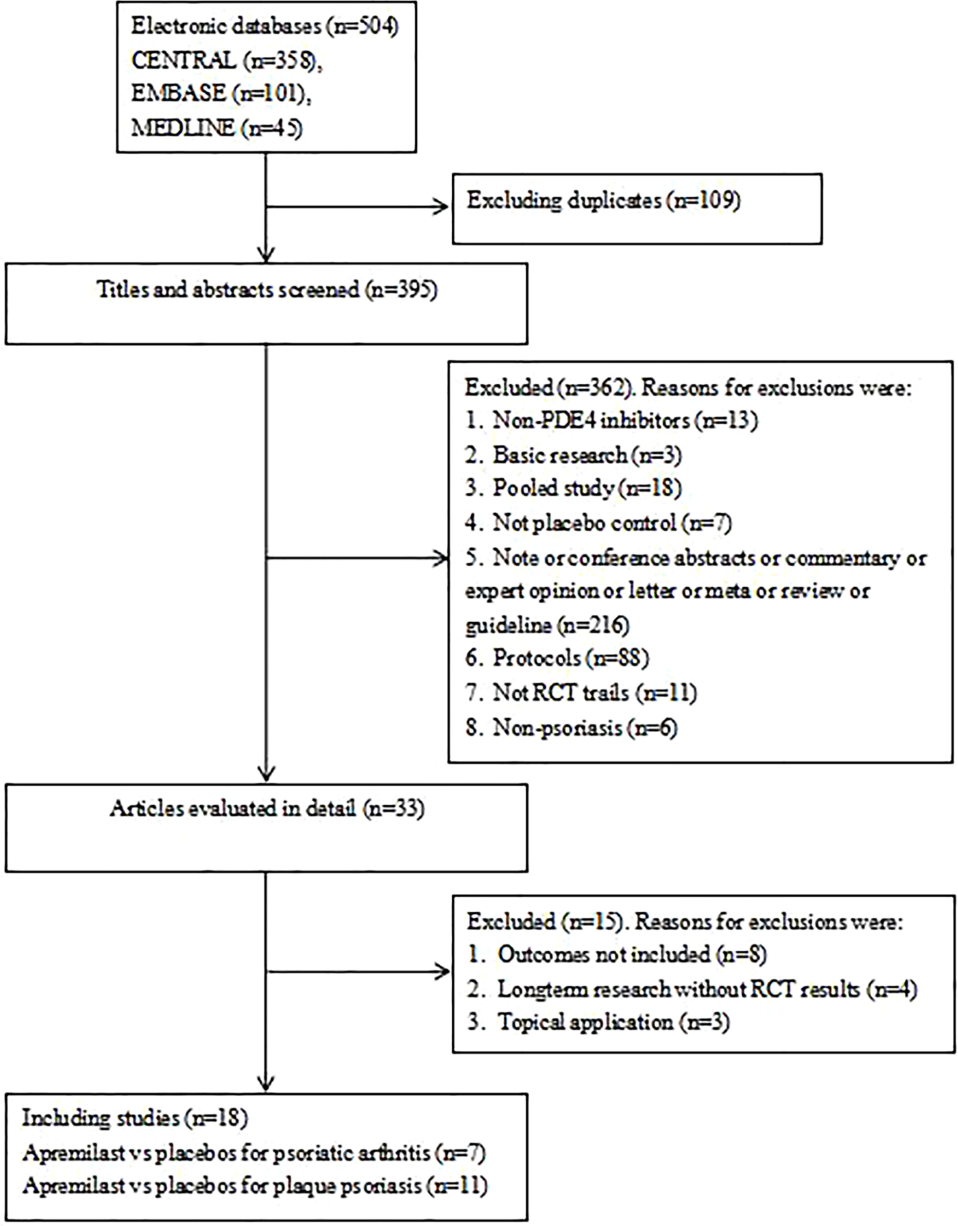
Flow of study identification, inclusion, and exclusion.

According to our search strategy, we initially identified 504 citations in three electronic databases (CENTRAL, MEDLINE, EMBASE). After excluding duplicated articles and screened titles and abstracts, we filtered out 33 RCTs that met the inclusion criteria. Finally, after further review of the full texts, we included 18 studies that could provide data for further analyses (**Figure 1**).

### Characteristics of clinical studies

A total of 6036 patients were included in this meta-analysis. Nine of the 18 studies included moderate-to-severe plaque psoriasis, 2 mild-to-moderate plaque psoriasis, and 7 arthropathy psoriasis. All patients were adults and received the same test drug, apremilast, but with different doses and duration of treatment. The study characteristics of these 18 selected trials are summarized in **Table 1** (15–32).

**Table 1:** Characteristics of the 18 included studies.

Study	Trial	No.of randomized participants (Age,y)	Type	Severity	Interventions	End point	Analyzed outcomes
Bissonnette 2018^15^	NCT02400749	100 (≧ 18)	Plaque psoriasis	moderate to severe	Apremilast 30mg bid	16 week	PPPGA, PPPASI-75, DLQI, PPPGA of 0 or 1, AEs
Cutolo 2016^16^	NCT01212757	484 (≧ 18)	Psoriatic Arthritis	**-**	Apremilast 20mg bid; 30mg bid	16 week	PASI-75, pain (VAS), AEs
Edwards 2016^17^	NCT01212770	505 (≧ 18)	Psoriatic Arthritis	**-**	Apremilast 20mg bid; 30mg bid	16 week 24 week	PASI-75, pain (VAS), AEs
Gold 2018^18^	NCT02425826	221 (≧ 18)	Plaque psoriasis	moderate	Apremilast 30mg bid	16 week	PASI-75, sPGA of 0 or 1, DLQI, pruritus (VAS), ScPGAof 0 or 1, AEs
Gold 2021^19^	NCT03721172	595 (≧ 18)	Plaque psoriasis	mild to moderate	Apremilast 30mg bid	16 week	sPGA of 0 or 1, DLQI, ScPGA of 0 or 1, AEs
Kavanaugh 2014^20^	NCT01172938	504 (≧ 18)	Psoriatic Arthritis	**-**	Apremilast 20mg bid; 30mg bid	16 week 24 week	PASI-75, pain (VAS), AEs
Nash 2018^21^	NCT01925768	219 (≧ 18)	Psoriatic Arthritis	**-**	Apremilast 30mg bid	16 week 24 week	AEs
Ohtsuki 2017^22^	NCT01988103	254 (≧ 20)	Plaque psoriasis	moderate to severe	Apremilast 20mg bid; 30mg bid	16 week	PASI-75, sPGA of 0 or 1, DLQI, pruritus (VAS), AEs
Papp 2012^23^	NCT00773734	352 (≧ 18)	Plaque psoriasis	moderate to severe	Apremilast 10mg bid; 20mg bid; 30mg bid	16 week	PASI-75, sPGA of 0 or 1, DLQI, AEs
Strand 2013-1^24^	NCT00773734	352 (≧ 18)	Plaque psoriasis	moderate to severe	Apremilast 10mg bid; 20mg bid; 30mg bid	16 week	Pruritus (VAS)
Papp 2013^25^	NCT00606450	260 (≧ 18)	Plaque psoriasis	moderate to severe	Apremilast 20mg Qd; 20mg bid	12 week	PASI-75, AEs
Papp 2015^26^	NCT01194219	844 (≧ 18)	Plaque psoriasis	moderate to severe	Apremilast 30mg bid	16 week	PASI-75, sPGA of 0 or 1,DLQI, pruritus (VAS), ScPGA of 0 or 1, NAPSI, AEs
Paul 2015^27^	NCT01232283	413 (≧ 18)	Plaque psoriasis	moderate to severe	Apremilast 30mg bid	16 week	PASI-75, sPGA of 0 or 1, DLQI, pruritus (VAS), ScPGA of 0 or 1, NAPSI, PPPGA of 0 or 1, AEs
Reich 2017^28^	NCT01690299	250 (≧ 18)	Plaque psoriasis	moderate to severe	Apremilast 30mg bid	16 week	PASI-75, sPGA of 0 or 1, DLQI, pain (VAS), pruritus (VAS), ScPGA of 0 or 1, NAPSI, AEs
Schett 2012^29^	NCT00456092	204 (≧ 18)	Psoriatic Arthritis	**-**	Apremilast 20mg bid; 40mg qd	12 week	DLQI, AEs
Strand 2013-2^30^	NCT00456092	204 (≧ 18)	Psoriatic Arthritis	**-**	Apremilast 20mg bid; 40mg qd	12 week	pain (VAS)
Van 2020^31^	NCT03123471	303 (≧ 18)	Plaque psoriasis	mild to moderate	Apremilast 30mg bid	16 week	DLQI, ScPGA of 0 or 1, AEs
Wells 2018^32^	NCT01307423	528 (≧ 18)	Psoriatic Arthritis	**-**	Apremilast 20mg bid; 30mg bid	16 week	PASI-75, pain (VAS), AEs

PASI, Psoriasis Area Severity Index; sPGA, Static Physician Global Assessment; ScPGA, Scalp Physician’s Global Assessment; PPPGA, Palmoplantar Psoriasis Physician Global Assessment; NPASI, Nail Psoriasis Severity Index; DLQI, Dermatology Life Quality Index; VAS, visual analogue scale; AE, adverse event.

### Overall clinical effects

#### Primary outcome

**Figure 2:**
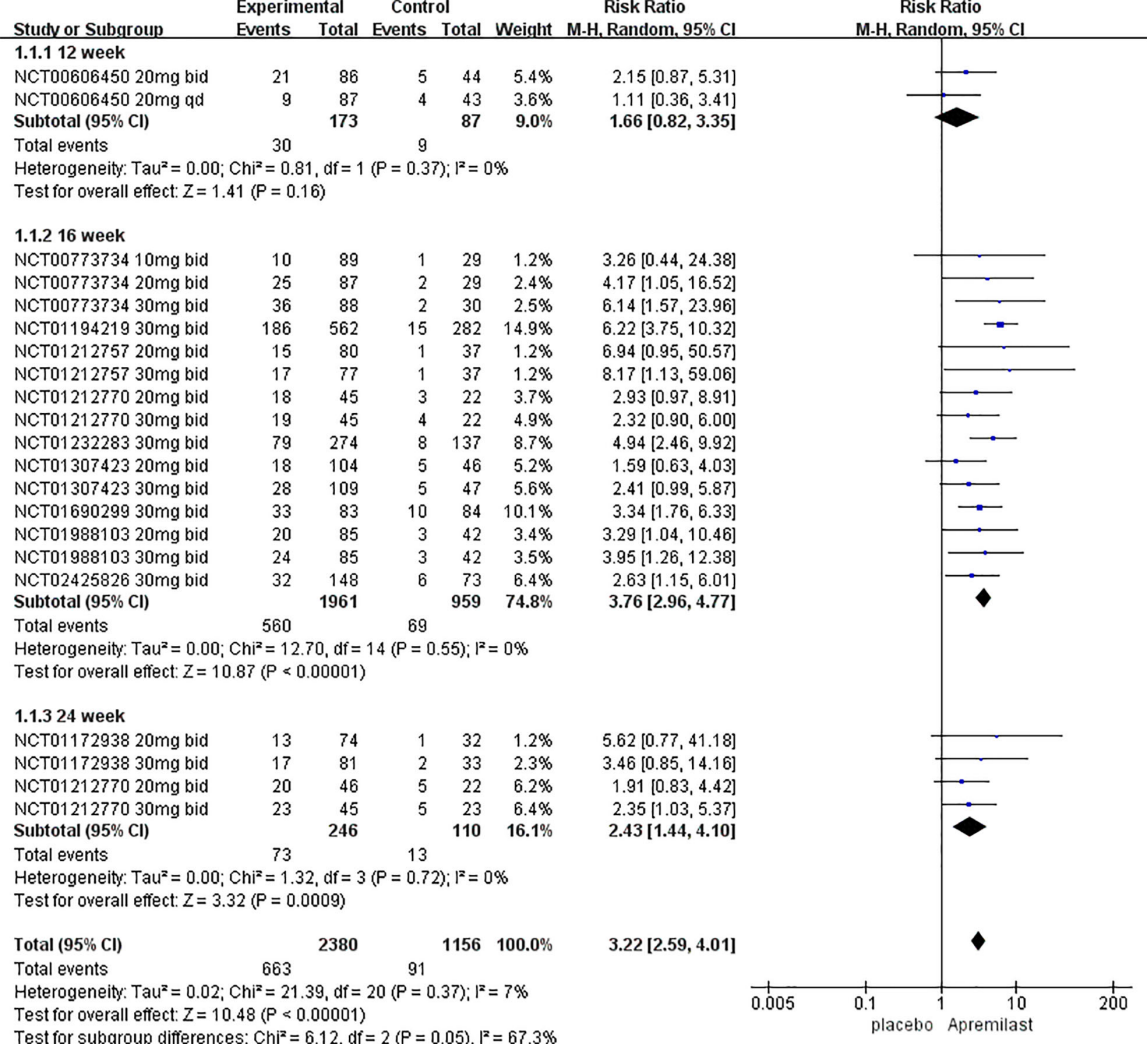
Meta-analysis of the association of phosphodiesterase 4 (PDE4) inhibitor vs placebos with the percentage improvement of PASI score by treatment duration.

We chose proportion of PASI-75 as the primary outcome. 11 trials including 3536 patients were analyzed. Overall, a random-effects model meta-analysis favored apremilast over placebo in terms of it (RR, 3.22; 95% CI, 2.59-4.01; P < 0.00001). Heterogeneity was not significant between studies (I^2^ = 7%; P = 0.37) (**Figure 2**).

#### Secondary outcomes

##### Percentage of participants with sPGA of 0 or 1

Seven trials including 2800 patients with plaque psoriasis were analyzed. Overall, compared with placebo, apremilast was associated with a significantly higher percentage of participants with sPGA of 0/1 (RR, 3.57; 95% CI, 2.58-4.93; P < 0.00001), and no significant heterogeneity between studies was detected (I^2^ = 26%; P = 0.20).

##### Improvement of patient’s subjective symptoms

In the assessment of pain symptom, 5 trials including 2166 patients with PsA analyzed improvement of joint pain, and one trial including 167 patients with plaque psoriasis analyzed improvement of skin pain. Of the 6 trials, 5 had a duration of 16 weeks and one had a 12-week duration. In the assessment of pruritus symptom, 6 trials including 2238 patients with plaque psoriasis analyzed the improvement in pruritus VAS at week 16. Overall, the pooled data found that apremilast was associated with a statistically significant decrease in pain VAS (SMD, -0.27; 95% CI, -0.36 to -0.18; P < 0.00001) and pruritus VAS (SMD, -0.51; 95% CI, -0.67 to -0.35; P < 0.00001) compared with placebo, respectively. However, significant heterogeneity between studies was detected in the analysis of pruritus score (I^2^ = 61%; P = 0.008), and no heterogeneity was detected in the analysis of pain score (I^2^ = 0%; P = 0.96).

##### Improvement of local lesions

The scalp, nails, palms, and soles are difficult-to-treat sites. Percentages of participants with ScPGA of 0/1, PPPGA of 0/1, and improvement of NAPSI were analyzed in 6 studies (including 2106 patients), 2 studies (including 142 patients), and 3 studies (including 1183 patients), respectively. All comparisons were apremilast 30 mg bid versus placebo at 16 weeks. The meta-analysis found that apremilast were associated with significantly higher scores in ScPGA of 0/1(RR, 2.21; 95% CI, 1.69-2.91; P < 0.00001), PPPGA of 0/1 (RR 2.33; 95%CI, 1.16-4.66; P =0.02) and significantly lower NAPSI (SMD, -0.46; 95% CI, -0.58 to -0.33; P < 0.00001). However, significant heterogeneity between studies was detected in ScPGA of 0/1 (I^2^ = 60%; P = 0.03), and no significant heterogeneity was detected in PPPGA of 0/1 (I^2^ = 0%; P = 0.55) or NAPSI (I^2^ = 1%; P = 0.37).

##### 4. Improvement of patients’ quality of life

10 studies including 3341 patients analyzed improvements in patients’ quality of life. The pooled data found that apremilast was associated with a statistically significant reduction in DLQI compared with placebo (SMD, -0.58; 95% CI, -0.65 to -0.50; P < 0.00001). Heterogeneity was not significant between studies (I^2^ = 3%; P = 0.42).

### Subgroup analysis

Subgroup analyses of PASI-75 were performed by pooling trials with the same clinical phenotype, dosage, and duration of treatment, respectively. **Table 2** shows that apremilast was associated with significantly higher response rates at the dosage of both 20 mg bid (RR, 2.82; 95% CI, 2.01 to 3.95; P < 0.00001) and 30 mg bid (RR, 4.08; 95% CI, 3.12 to 5.33; P < 0.00001), treatment course of both 16 weeks (RR, 3.78; 95% CI, 2.97 to 4.81; P < 0.00001) and 24weeks (RR, 2.47; 95% CI, 1.41 to 4.31; P=0.002), and clinical phenotype of plaque psoriasis (RR, 3.67; 95% CI, 2.72 to 4.97; P< 0.00001) and PsA (RR, 2.48; 95% CI, 1.76-3.50; P< 0.00001). However, at week 12 (RR, 1.66; 95% CI,0.82-3.35; P=0.16), dosage of 10 mg bid (RR, 1.98; 95% CI, 0.70-5.50; P=0.20) and 20mg qd (RR, 1.00; 95% CI, 0.42-2.40; P=1.00), no significant differences in PASI-75 were found between the apremilast groups and the placebo groups. Pooled studies across all subgroups were homogenous (I^2^<50%; P>0.1).

**Table 2:** Subgroup analyses of randomized clinical trials of apremilast for PASI-75 in psoriasis.

Factor	Trails, No.	Apremilast/placeboParticipants, No.	RR (95% CI)	P value	I^2^, %;P value
**Therapeutic Dose**					
10mg bid	1	89/88	1.98 (0.70 to 5.55)	0.20	NA
20mg bid	7	607/580	2.82 (2.01 to 3.95)	< 0.00001	3; 0.40
30mg bid	10	1597/1069	4.08 (3.12 to 5.33)	< 0.00001	17; 0.29
20mg qd	1	87/87	1.00 (0.42 to 2.40)	1.00	NA
**Treatment Course**					
12 weeks	1	173/87	1.66 (0.82 to 3.35)	0.16	NA
16 weeks	9	2052/1004	3.78 (2.97 to 4.81)	< 0.00001	0; 0.57
24 weeks	2	336/154	2.47 (1.41 to 4.31)	0.002	0; 0.75
**Clinical Phenotype**					
PsA with plaque psoriasis	4	706/321	2.48 (1.76 to 3.50)	< 0.00001	0; 0.84
plaque psoriasis	7	1674/835	3.67 (2.72 to 4.97)	< 0.00001	18; 0.27

NA, not applicable; PsA, psoriatic arthritis; RR, relative risk.

### Safety outcomes

#### TEAEs Leading to Withdrawal

TEAEs led to study withdrawal of participants in 14 trials, including 283 patients. The results showed that apremilast was associated with a higher proportion of patients with TEAEs leading to withdrawal (RR, 1.41; 95% CI, 1.09-1.83; P=0.009), with no heterogeneity between studies (I^2^ = 0%; P =1.00).

#### At least 1 TEAE

15 trials including 3454 patients reported at least one TEAE. The pooled data showed that apremilast was associated with a higher proportion of patients with at least 1 TEAE (RR, 1.22; 95% CI, 1.17-1.28; P<0.00001), with no heterogeneity between studies (I^2^ = 0%; P =0.62).

#### At least 1 serious TEAE

There was no statistically difference in the results between apremilast and placebo treatments (RR,0.80; 95% CI, 0.56-1.15; P = 0.23). In these studies, 133 of 5839 patients had at least 1 serious TEAE, with apremilast group (82 of 3759 [2.18%]) and placebo group (51 of 2080 [2.45%]) were similar in incidence. No heterogeneity was detected (I^2^ = 0%; P = 0.86).

#### Any drug-related TEAEs

13 trials including 5228 patients reported on drug-related TEAEs. Results showed that compared with placebo (328 of 1905 [17.2%]), apremilast treatment (1156 of 3323 [34.79%]) was associated with a significantly higher incidence of any drug-related TEAEs (RR, 1.98; 95% CI, 1.77-2.20; P < 0.00001), and no heterogeneity was detected (I^2^ = 0%; P = 0.82). Drug-related TEAEs in the apremilast group included diarrhea, nausea, depression, not serious suicidal ideation, weight loss, dizziness, decreased blood pressure, sinusitis, hepatic enzyme increase, headache, and migraine. These TEAEs were reported to be mild to moderate, with some resolving after discontinuation and some resolving within 1 month despite continued treatment and no pharmacological intervention. Some serious apremilast-related TEAEs have been reported, including abdominal abscess, diverticulitis, pneumonia and gastrointestinal clostridial infection. However, the only reported event of diverticulitis resolved without any does change and medicinal intervention. The patients with pneumonia and gastrointestinal clostridial infections recovered after standard course of antibiotic therapy. Other serious adverse events, including malignant diseases, systemic vasculitis, major cardiovascular events, or death, were not considered treatment-related.

### Risk of bias and publication bias assessment

The included trials were determined to have a low and unclear risk of bias (**eFigure 1** in the Supplement). For the outcome of proportion of PASI-75, no publication bias was detected by using an Egger’s test (bias, -0.98; 95% CI, -5.74 to 3.78; p = 0.67) or Begg’s test (pr> |z|=0.239).

### Sensitivity analyses

In the pooled data on pruritus VAS improvement, we found that the heterogeneity came from trial NCT01194219. After excluding this study, no significant heterogeneity was detected (I^2^ = 30%; P = 0.19), and the effect on decreasing pruritus score was statistically unchanged in the meta-analysis (SMD, -0.46; 95% CI, -0.60 to -0.32; P < 0.00001). In meta-analysis of ScPGA of 0/1, we found that the heterogeneity came from trial NCT02425826, and after excluding this study, no heterogeneity was detected (I^2^ = 0%; P = 0.56), and the effect on the proportion of ScPGA of 0/1 was unchanged statistically (RR, 2.52; 95% CI, 2.09 to 3.04; P < 0.00001).

## Discussion

To our knowledge, this is the first study to systematically analyze the efficacy and safety of PDE4 inhibitor for the treatment of both PsA and plaque psoriasis using RCTs. Ultimately, only one oral PDE4 inhibitor, apremilast, met the inclusion criteria. Overall, the results of this meta-analysis of 18 studies showed that apremilast was effective in improving skin lesions and quality of life in patients with psoriasis, both PsA and plaque psoriasis, especially after 16 weeks of treatment. And apremilast was well tolerated.

PASI-75 is a classic tool for assessing the efficacy of psoriasis treatment (33). And 68.75% (11/16) of the trials included in this meta-analysis evaluated PASI-75, which is the most described tool in the trials included in this study. Therefore, we chose the proportion of PASI-75 as the primary outcome of this meta-analysis. In addition, sPGA of clear or almost clear skin was evaluated in 7/16 (43.75%) of included trials. Both PASI and sPGA are measures of general physical signs. Across all the included trials, apremilast significantly improved general physical signs in patients with psoriasis compared with placebo.

However, PASI and sPGA do not adequately reflect the severity of local lesions, such as scalp, nails, palms, and soles. These parts are more common at the onset of psoriasis and throughout the course of the disease (34), and are difficult to hide, which seriously affects the patients’ self-esteem and social ability (35). And worst of all, the treatments of psoriasis in these specific areas are very challenging, topical therapies are often ineffective (34, 36–38). Therefore, effective systemic treatment is required. Our study shows that apremilast can significantly improve all of these local signs. In addition, from a patient’s perspective, apremilast significantly reduced itching and pain and improved quality of life. Our conclusions are consistent with previous pooled studies (39, 40) and real-world findings (41, 42).

No publishing bias was detected in the measurement of PASI-75, and no significant heterogeneity was detected in the overall comparative study between apremilast and placebo. Although heterogeneity between studies was significantly higher for ScPGA 0/1 (I^2 =^ 60%, p = 0.03) and pruritus scores (I^2 =^ 61%; p=0.008), sensitivity analysis found that no effect estimate was statistically changed after excluding the source studies of heterogeneity. These findings suggest that the efficacy of apremilast in the treatment of psoriasis is robust and reproducible. Therefore, apremilast represents a new option for psoriasis therapy.

Furthermore, we found that apremilast 20 mg bid and 30 mg bid were more effective, while 10 mg bid and 20 mg qd were similarly effective with placebo, suggesting that the efficacy of apremilast was dose-dependent. Based on time-to-treatment analysis, the optimal treatment duration for apremilast to improve PASI of psoriasis was ≥16 weeks, suggesting that apremilast does not provide rapid relief of psoriatic symptoms. Therefore, apremilast is not suitable for monotherapy in critically ill patients who require rapid improvement of clinical symptoms and quality of life.

The safety analysis showed that while apremilast had higher rates of any TEAEs, drug-related TEAEs and withdrawal due to TEAEs than placebo, there were no significant differences in serious TEAEs, such as opportunistic infections, malignant disease, systemic vasculitis, major cardiovascular events, or death. In addition, although the incidence of drug-related TAEAs in apremilast group (34.79%) was almost double that in the placebo group (17.2%), most were mild to moderate, and most were self-limited or resolved after drug discontinuation. Infections were the most frequently reported serious TEAEs, including abdominal abscess, diverticulitis, pneumonia, and gastrointestinal infections, which either resolved spontaneously without intervention or with standard anti-infective therapy. In general, our study suggests that apremilast is well tolerated.

## Limitations

Available data were limited to the assessment of PPPGA 0/1, in which less than 200 patients were tested. The higher treatment goals of PASI-90 or PASI-100, which were not evaluated in this study, are another limitation. Furthermore, there are limited data available for subgroup analyses based on geography or ethnicity. Body weight or body surface area greatly affects the efficacy of systemic therapy (43). Asians are generally smaller in weight or body surface area than Europeans and Americans. However, there was only one study in the Japanese population in our study, the rest were from Europe and the Americas. In addition, the long-term efficacy, retention rate, cost and comparison with other drugs of apremilast in the treatment of psoriasis also need further study.

## Conclusions

The results of this meta-analysis support the favorable efficacy and safety profile of apremilast in the treatment of plaque psoriasis and PsA, especially in difficult-to-treat areas. More studies are needed in the future in Asian populations and in difficult-to-treat areas such as nails, scalp, palms, and soles, as well as evaluation with higher goals, such as PASI 100.

## Data availability statement

The raw data supporting the conclusions of this article will be made available by the authors, without undue reservation.

## Author contributions

HY had the conception, collected and analyzed the data, wrote the manuscript, and revised the manuscript. QK collected and analyzed the data, and wrote the manuscript. J-sC collected and analyzed the data, and helped in the methods. All authors contributed to the article and approved the submitted version.

## Funding

This work was supported by the joint project of Chongqing Municipal Health Commission and Chongqing Municipal Science and Technology Bureau (No. 2020MSXM070 to HY), and the second batch of Class A reserve talents of the Children’s Hospital Affiliated to Chongqing Medical University (No. RC05036 to HY).

## Conflict of interest

The authors declare that the research was conducted in the absence of any commercial or financial relationships that could be construed as a potential conflict of interest.

## Publisher’s note

All claims expressed in this article are solely those of the authors and do not necessarily represent those of their affiliated organizations, or those of the publisher, the editors and the reviewers. Any product that may be evaluated in this article, or claim that may be made by its manufacturer, is not guaranteed or endorsed by the publisher.

## Supplementary material

Supplementary Figure 1 Assessment of risk bias of RCTs.
