# Supplementary figures and images for: Efficacy and safety profile of phosphodiesterase 4 inhibitor in the treatment of psoriasis: A systematic review and meta-analysis of randomized controlled trials
Source: Front Immunol. 2022 Oct 10;13:1021537. doi: 10.3389/fimmu.2022.1021537 (PMC9589065; doi:10.3389/fimmu.2022.1021537)

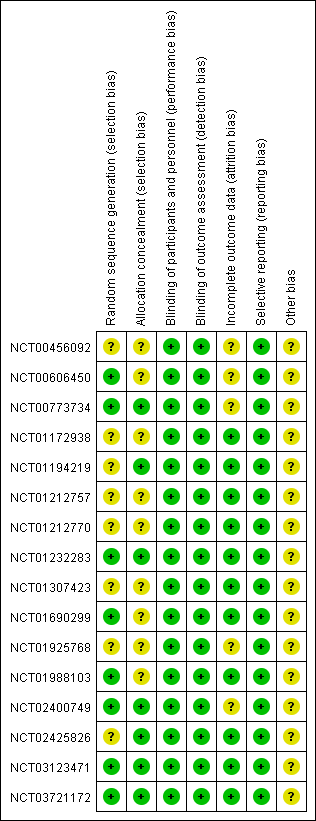

Supplement: Supplementary Figure 1 — Assessment of risk bias of RCTs. [file Image_1.jpeg]
