# Supplementary material for: Efficacy and safety profile of phosphodiesterase 4 inhibitor in the treatment of psoriasis: A systematic review and meta-analysis of randomized controlled trials
Source: Front Immunol. 2022 Oct 10;13:1021537. doi: 10.3389/fimmu.2022.1021537 (PMC9589065; doi:10.3389/fimmu.2022.1021537)
Supplement: Supplementary file 2 [file Table_1.docx]

eTable 1. Search strategy in CENTRAL, EMBASE and MEDLINE (n = 504)

1. Search strategy used in CENTRAL (n = 358)

#1 (psoriasis) (Word variations have been searched) 8994

#2 Phosphodiesterase 4 Inhibitor 1355

#3 PDE4 inhibitor 191

#4 PDE-4 inhibitor 44

#5 Hemay005 8

#6 Apremilast 516

#7 Roflumilast 425

#8 ARQ-151 18

#9 AN2728 21

#10 #2 OR #3 OR #4 OR #5 OR #6 OR #7 OR #8 OR #9 1999

#11 #1 AND #10 368

#12 Trials 358

1. Search strategy used in EMBASE (n = 101)

#1. 'psoriasis'/exp OR psoriasis 114,221

#2. 'phosphodiesterase 4 inhibitor' 1,080

#3. 'phosphodiesterase iv inhibitor' 3,273

#4. 'pde4 inhibitor' 1,466

#5. 'pde-4 inhibitor' 231

#6. hemay005 1

#7. 'apremilast' 3,181

#8. 'roflumilast' 2,321

#9. 'arq 151' 7

#10. an2728 49

#11. #2 OR #3 OR #4 OR #5 OR #6 OR #7 OR #8 OR #9 OR #10 8,497

#12. 'randomized controlled trial' 964,423

#13. 'randomization' 127,500

#14. 'controlled clinical trial' 464,739

#15. #12 OR #13 OR #14 1,233,324

#16. 'letter to the editor' 28,842

#17. 'conferences and congresses' 844

#18. conference 5,345,499

#19. 'case report form' 1,923

#20. 'review' 5,591,810

#21. #16 OR #17 OR #18 OR #19 OR #20 10,507,542

#22. #15 NOT #21 777,748

#23. #1 AND #11 AND #22 101

1. Search strategy used in MEDLINE (n = 45)

|  | **Searches** | **Results** |
| --- | --- | --- |
| 1 | psoriasis.mp. or exp Psoriasis/ | 57973 |
| 2 | phosphodiesterase 4 inhibitor.mp. or exp Phosphodiesterase 4 Inhibitors/ | 2811 |
| 3 | PDE4 inhibitor.mp. | 906 |
| 4 | PDE-4 inhibitor.mp. | 129 |
| 5 | Hemay005.mp. | 1 |
| 6 | Apremilast.mp. | 912 |
| 7 | Roflumilast.mp. | 717 |
| 8 | ARQ-151.mp. | 3 |
| 9 | AN2728.mp. | 8 |
| 10 | 2 or 3 or 4 or 5 or 6 or 7 or 8 or 9 | 4026 |
| 11 | Randomized Controlled Trial.mp. or exp Randomized Controlled Trial/ | 609227 |
| 12 | Random Allocation.mp. or exp Random Allocation/ | 108622 |
| 13 | Randomly allocated.mp. | 33380 |
| 14 | Controlled Clinical Trial.mp. or exp Controlled Clinical Trial/ | 668821 |
| 15 | 11 or 12 or 13 or 14 | 798636 |
| 16 | letter.mp. or exp Letter/ | 1241216 |
| 17 | exp "Utilization Review"/ or exp Peer Review, Health Care/ or exp "Peer Review"/ or exp "Systematic Review"/ or exp "Review"/ or review.mp. or exp "Rate Setting and Review"/ or exp "Medication Review"/ or exp "Scientific Integrity Review"/ or exp "Review Literature as Topic"/ or exp "Concurrent Review"/ or exp "Physician Payment Review Commission"/ or exp "Professional Review Organizations"/ or exp Peer Review, Research/ or exp "Ethical Review"/ or exp "Drug Utilization Review"/ or exp "Insurance Claim Review"/ | 3730759 |
| 18 | conference.mp. or exp Congresses as Topic/ | 109777 |
| 19 | case report.mp. or exp Case Reports/ | 2330018 |
| 20 | 16 or 17 or 18 or 19 | 6892605 |
| 21 | 1 and 10 and 15 | 53 |
| 22 | 21 not 20 | 45 |
